# Naturalistic rapid deceleration data: Drivers aged 75 years and older

**DOI:** 10.1016/j.dib.2016.10.024

**Published:** 2016-11-02

**Authors:** Anna Chevalier, Aran John Chevalier, Elizabeth Clarke, Kristy Coxon, Julie Brown, Kris Rogers, Soufiane Boufous, Rebecca Ivers, Lisa Keay

**Affiliations:** a The George Institute for Global Health, Sydney Medical School, The University of Sydney, PO Box M201, Missenden Rd, Sydney, NSW 2050, Australia; b Safer Roads Consulting, 53 Lachlan St, Thirroul, NSW 2515, Australia; c Kolling Institute of Medical Research, Sydney Medical School, The University of Sydney, Level 10, Kolling Building 6, Royal North Shore Hospital, St Leonards, NSW 2065, Australia; d School of Science and Health, Western Sydney University, Narellan Road, Campbelltown, NSW 2560, Australia; e Neuroscience Research Australia (NeuRA), Margarete Ainsworth Building, Barker St, Randwick, NSW 2031, Australia; f Transport and Road Safety Research (TARS), Level 1, West Wing, Old Main Building, University of NSW, Sydney, NSW 2052, Australia

**Keywords:** Deceleration, Naturalistic, Older drivers, Surrogate safety measure

## Abstract

The data presented in this article are related to the research manuscript “Predictors of older drivers’ involvement in rapid deceleration events”, which investigates potential predictors of older drivers’ involvement in rapid deceleration events including measures of vision, cognitive function and driving confidence (A. Chevalier et al., 2016) [1]. In naturalistic driving studies such as this, when sample size is not large enough to allow crashes to be used to investigate driver safety, rapid deceleration events may be used as a surrogate safety measure. Naturalistic driving data were collected for up to 52 weeks from 182 volunteer drivers aged 75–94 years (median 80 years, 52% male) living in the suburban outskirts of Sydney. Driving data were collected using an in-vehicle monitoring device. Accelerometer data were recorded 32 times per second and Global Positioning System (GPS) data each second. To measure rapid deceleration behavior, rapid deceleration events (RDEs) were defined as having at least one data point at or above the deceleration threshold of 750 milli-g (7.35 m/s^2^). All events were constrained to a maximum 5 s duration. The dataset provided with this article contains 473 events, with a row per RDE. This article also contains information about data processing, treatment and quality control. The methods and data presented here may assist with planning and analysis of future studies into rapid deceleration behaviour using in-vehicle monitoring.

**Specifications Table**

**Table:** 

Subject area	Road safety
More specific subject area	Rapid deceleration; older drivers
Type of data	Table of data, table describing variables, two figures
How data were acquired	The in-vehicle monitoring device consisted of a C4D Data Recorder with an external GPS receiver. The hardware included an internal tri-axial accelerometer, tachograph, real-time clock, 128 MB of flash memory and internal battery (1300 mA). Accelerometer data were recorded at 32 Hz (32 times per second). The GPS data were recorded at 1 Hz (each second).
Data format	Processed, assessed for quality control
Experimental factors	Accelerometer data were treated to address calibration and wandering baseline.
Experimental features	Data collection, the definition developed for rapid deceleration events and steps taken to process data to identify and validate these events are detailed below.
Data source location	North-west Sydney.
Data accessibility	The dataset is within this article.

**Value of the data**

• Naturalistic methods are being used increasingly in road safety research, but little is known about the distribution of this type of data. The data provided in this manuscript may be used to calculate sample sizes for other studies investigating rapid deceleration behavior.
• Methodological considerations are reported including treating accelerometer data to address calibration and wandering baseline issues, monitoring inactivity, and quality control.
• This data could be considering in future meta-analysis combining this data about older drivers’ deceleration behavior with other datasets which include a broader range of age groups and other settings.

## 1 Data

**Fig. 1:**
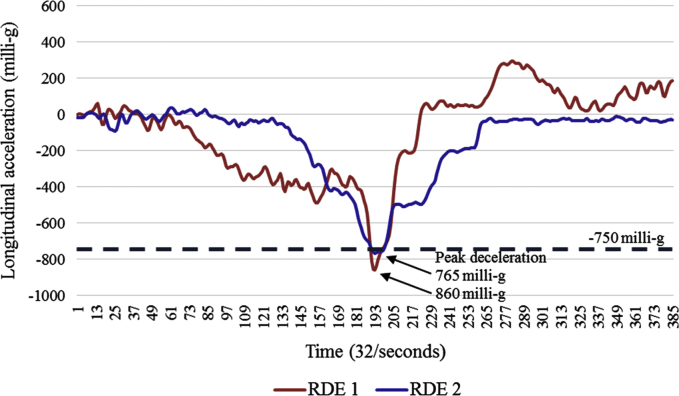
Two >750 milli-g rapid deceleration events during a week of one participant׳s driving.

**Fig. 2:**
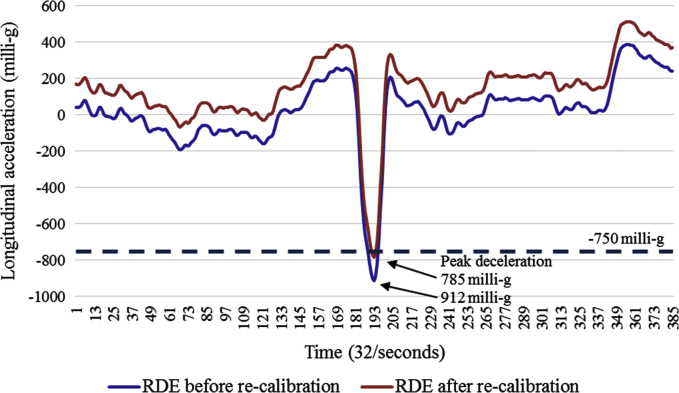
Single >750 milli-g rapid deceleration event before and after applying re-calibration adjustment.

The dataset contains one row per rapid deceleration event (Supplementary Table 1). The variables within the dataset are described in Table 1. Fig. 1 depicts two rapid deceleration events during a week of one participant׳s driving, and Fig. 2 depicts accelerometer recordings during a RDE before and after a re-calibration adjustment was applied.

## 2 Experimental design, materials and methods

### 2.1 Participants

Volunteer participants from the control group of a randomised control trial (*n*=380) [2] agreed to have their vehicle instrumented (*n*=182/190) for up to 12 months. Participants were aged 75–94 years (median 80 years) and 52% (95/182) were male. Participants resided in the urban outskirts of north-west Sydney (in the Hills, Hornsby, Kur-ring-gai and Parramatta Local Government Areas); held a driver׳s licence; owned and were the primary driver of a vehicle (undertaking greater than 80% of driving). Participants were excluded if they received greater than two errors on the Short Portable Mental Status Questionnaire cognitive assessment [3]. Data were collected between July 2012 and May 2014. The GPS derived speed and exposure data have been linked to demographic information about participants in other analyses [4], [5], [6], [7], [8].

#### 2.1.1 Data acquisition

The C4D Data Recorder was approximately 11 cm × 8.5 cm × 3 cm in size with an external GPS receiver. The hardware included an internal tri-axial accelerometer, tachograph, real-time clock, 128 MB of flash memory and internal battery (1300 mA). Accelerometer data are based on a three-dimensional Cartesian coordinate system, capturing longitudinal (x-axis), lateral (y-axis) and vertical acceleration (z-axis). To enable the accelerometer to record accurate measurements, devices were installed aligned with direction of travel, with a specified orientation and positioned as flat as possible, with the vehicle on as flat ground as possible. The devices were secured in a concealed area either under the dash, fan or front passenger seat, using one or a combination of silicone, double sided tape and cable ties.

A custom-designed cable integrated the unit into the vehicle electrical system, providing a fused connection to the ignition and electrically grounding the vehicle. The device powered on and off with the vehicle ignition. The device transmitted captured data to a secure service provider server via the mobile telecommunications network every 20 s while the vehicle was running. The provider uploaded data each week to the researcher׳s secure server, and to protect data confidentiality, both servers restricted staff access.

Accelerometers are used for many applications, including by vehicle manufacturers to deploy airbags in the event of an impending crash, and in smart phones and laptops to protect the device in the event of a fall by initiating shut down. The study accelerometer had a maximum capacity of 2000 milli-g (where milli-g indicates one thousandth of the acceleration due to gravity (approx. 0.0098 m/s^2^)), with a buffer of approximately 250 milli-g, and a precision of 18 milli-g. While this did not allow for capturing peak decelerations experienced in the event of a high-impact crash, limiting the range of detectable acceleration increased the level of precision.

As the accelerometer x, y and z coordinates were not pre-calibrated by the international supplier, the local service provider calibrated each axis at installation. The service provider collected device data at installation while the ignition was running and the vehicle was at a standstill. Approximately one second worth of these values (30 recordings) were used to calculate the mode for each axis, and these offsets were subtracted to re-zero each axis. These offsets were set each time a device was installed.

Weekly datasets included for each participant trip a (i) non-aggregated file of accelerometer data recorded at 32 Hz, (ii) non-aggregated file of GPS data recorded at 1 Hz, and (iii) mapped kmz format file. The kmz files opened in Google Earth [9] and depicted the non-aggregated GPS data for each participant trip, including start and end locations, route driven and direction of travel. These data were used to manually examine validity of individual trip data where necessary.

### 2.2 Rapid Deceleration Event (RDE) definition

As described in the related research manuscript [1], a RDE was defined as having at least one data point (approximately a 32nd of a second) at or above the deceleration threshold of 750 milli-g (Fig. 1). The highest deceleration figure was identified for the event. To minimise the chance of identifying false positive events, RDEs that were likely to be related to a single incident were counted as a single event, defined as occurring within 3 s of the previous event dropping below 500 milli-g deceleration and the beginning of the next event being >=500 milli-g deceleration, and each event having a peak of >=500 milli-g and at least one with a peak of >750 milli-g. For example, if a driver released and then re-applied the brake to decelerate at >=500 milli-g it would be counted as a single RDE. The 3 s is based on advisory best practice for safe vehicle following distance (headway) [10]. The longest individual RDE duration was 4.5 s from >=500 milli-g deceleration to peak of >750 milli-g deceleration and returning to >=500 milli-g deceleration.

In our trial protocol, we originally specified 500 milli-g and 1000 milli-g [2] as deceleration thresholds for defining RDEs. However, within our cohort >500 milli-g RDEs were very common, with 11,354 identified during the period of monitoring (ranging up to 405 per participant, median=38, IQR=16–95). Therefore, we applied a >750 milli-g threshold (mid-way between the two pre-specified thresholds), as this represents a substantial deceleration and should increase specificity of these events. Though we were are unable to confirm events using video recording of the roadway, research indicates braking harder than 600 milli-g is infrequent [11]. A deceleration of 750 milli-g for one second would slow a vehicle by 26.5 km/hr. A deceleration of 486 milli-g has been described as “…the driver would feel like they were thrown forward towards the steering wheel and the load in the vehicle would shift to the front of the vehicle. Objects placed on the seat unrestrained would probably be thrown to the floor.” [12]. A forward collision is likely to measure several thousand milli-g or more of deceleration.

### 2.3 Device malfunctions

To identify device malfunctions, inactivity was monitored approximately every three days. The service provider sent inactive devices a Short Message Service (SMS) and asked the device to respond. To reduce the number of phone calls to infrequent drivers, the participant was only contacted by the study team if the device was inactive for 14 days. During a typical month, approximately 2% (2/114) of installed control group devices were identified as inactive and 8 inactivity tests were conducted. In the majority of cases, the participant reported not driving (due to vacation, illness, rain, injury or vehicle breakdown). If a device was identified as not recording driving (inactivity recorded but participant reported that they were still driving), a technician made a maintenance visit to fix or replace the device, which only occurred approximately 8 times for the 182 participants during our study. Where a participant may have been driving or staying in a remote area without General Packet Radio Service (GPRS) coverage, data would be stored on the device and uploaded to the server upon their return to an area with coverage.

### 2.4 Processing data

Due to the large volume of data, robust programs were required to maintain the integrity, and confirm the validity of the data by (i) ensuring each file was attributed to the correct device, participant and date; (ii) thoroughly checking data as processed to identify errors such as missing files and missing rows of data; and (iii) automating processing to reduce the need for manual handling of data, processing time and incidence of human error.

Data were provided for all participants by project week. Matlab [13] programs were developed to copy these data into program-created folders for individual participants by their week of involvement in the study (participant week). To identify the relevant participant weeks, the program referred to a mapping csv file containing unique participant and device identifiers, as well as device installation dates. The programs identified when a participant had more than one device during a single week, identified the order of device installation, and re-numbered trips for the week sequentially. Of the 182 participants, 20 had two devices and one had three devices installed in their vehicle/s during the study.

### 2.5 Noise

As part of processing the accelerometer data to identify RDEs, a low pass Butterworth filter with order 2 and a cut off frequency of 3 Hz was applied within a Matlab [13] program to smooth the data [14]. This filter was confirmed appropriate for detecting known positive RDEs using accelerometer data captured during low-speed crash sled testing [14] and evaluation of known positive events in a pilot vehicle. In addition, as described in the related research manuscript [1], the Butterworth filter used to smooth the accelerometer data applied a non-linear phase filter causing differential delay across the frequency spectrum. While we are not aware that this affected the identification of RDEs, an improved methodology in future studies to avoid this would be to apply a linear phase FIR filter for offline processing.

### 2.6 Accelerometer data calibration

Based on the assumption that over the course of a trip, acceleration and deceleration should roughly have an average close to zero (assuming gravitational force maintains a fairly constant 1 g (1000 milli-g)), the accelerometer data were treated for two calibration issues. A device in one or a combination of the 3 axes may have had (i) an inaccurate baseline calibration, and/or (ii) the baseline may have wandered once or more over time.

To address these issues, the average x-axis accelerometer for each trip was subtracted from each record within the trip. To address the possibility of large re-calibrations indicating an unreliable device, a decision was made *a priori* to exclude RDEs with a trip mean of greater than plus or minus 150 milli-g in any of the three axes.

To confirm this re-calibration method was effective, a manual audit compared RDEs between untreated and treated data from 20 randomly selected participant weeks. This analysis was based on RDEs with a 500+ milli-g deceleration threshold. Random sampling was undertaken in Excel to determine which participant weeks were included within this audit. The audit found the data to be comparable, with the x-axis centred around zero. When compared to the not re-calibrated data, only a very small number of RDEs not identified were due to inaccurate calibration or wandering baseline.

Applying the re-calibration adjustment resulted in small corrections to the accelerometer data. Of the 473 identified >750 milli-g deceleration threshold RDEs, the mean trip x-axis offset was 5 milli-g (SD 52.3, min 131, max −142). The mean trip z-axis offset was −1 milli-g (SD 17, min 35, max −79), and y-axis offset was 1 milli-g (SD 41, min 108, max −141). As at the >750 milli-g threshold, no trip means greater than ±150 milli-g in any of the three axes were identified, no RDEs were excluded on this basis. Fig. 2 depicts the accelerometer recordings during a >750 milli-g RDE before and after a re-calibration adjustment of −127 milli-g was applied.

As described in the related research manuscript [1], while our device did not have an electronic inclinator with a level coordinate system, this would have been useful to measure tilt within the devices, to ensure the device was mounted flat and facing precisely the correct directions. Inclinator measurements could be used to incrementally adjust the effect of the z-axis out of the x and y readings. This would be particularly useful for treating changes in the x-axis readings over time, and to accurately account for changes in gradient (ie hills, valleys, steep driveways, etc).

### 2.7 Data quality control

Where a participant reported they had lent their vehicle to a relative or friend, data for this period were replaced with missing values. Data from malfunctioning devices were also replaced with missing values.

We thoroughly examined the accelerometer data to confirm validity and that RDE definition parameters were met. RDE data validity criteria were developed for individual events and aggregated weekly event data. Examination of raw data found instances where duration was a negative value were caused by device malfunctions related to the accelerometer time incorrectly recorded by the device jumping forward and backward in time or a fault with the device powering off. Individual 500+ milli-g RDEs with a duration of minus seconds were excluded from analysis. Aggregated RDEs per participant week were excluded if they contained a single accelerometer trip file size >50 MB/trip or combined accelerometer trip file sizes >200 MB/week, which indicated a device malfunction by not powering off with the vehicle. Data from many RDEs were individually inspected, including those with the highest decelerations. After undertaking data quality control, all 473 RDEs >750 milli-g were considered valid. Some participants were involved in a few or several RDEs within a short period of time. For example, participant 1135 in his/her 22nd week of monitoring was involved in 14 RDEs within 25 min, and participant 1365 in his/her first week of monitoring was involved in 7 RDEs within 4 min. Presumably these were recorded during periods of difficult driving (ie negotiating steep hills, mountain passes or tight curves). Both of these participants were also involved in RDEs on other days, with 1135 involved in the highest number of RDEs/participant (28 RDEs over the period of monitoring).

During monitoring, 64% (114/177) of participants were involved in at least one of the 473 RDEs recorded. Applying a negative binomial model in SAS without exposure variables, the dispersion factor was 1.72 when no offset was applied, 1.69 when duration of monitoring was applied as an offset, and 1.93 when distance driven was applied as an offset [15]. This information may be useful to those planning sample sizes for studies of older drivers involved in RDEs.

## Figures and Tables

**Table 1:** Description of variables in the dataset of validated rapid deceleration events.

Variable name	Description
Partid	Unique participant identification number
Partweekno	Week number in the study (1–52 weeks)
Rdeno_valid	Event number
Peakdeceleration	Maximum deceleration during event (event peak) (milli-g), with higher values indicating greater deceleration
Date	Date (yyyymmdd) (derived from accelerometer Unix timestamp)
Time	Time at event peak (hh:mm:ss, 24 h) (derived from accelerometer Unix timestamp)
